# Polyhedral Oligomeric Silsesquioxane (POSS) Surface Grafting: A Novel Method to Enhance Polylactide Hydrolysis Resistance

**DOI:** 10.3390/nano9081144

**Published:** 2019-08-09

**Authors:** Kun Li, Samuele Colonna, Alberto Fina, Orietta Monticelli

**Affiliations:** 1 Dipartimento di Chimica e Chimica Industriale, Università di Genova, Via Dodecaneso, 31, 16146 Genova, Italy; 2 Dipartimento di Scienza Applicata e Tecnologia, Politecnico di Torino- Alessandria campus, viale Teresa Michel, 5, 15121 Alessandria, Italy

**Keywords:** Polylactide (PLA), hydrolytic degradation, polyhedral oligomeric silsesquioxane (POSS), surface grafting

## Abstract

This work considers the development of an easy and scalable approach to change the features of poly(l-lactide) (PLLA) films, which is based on the application of a surface treatment with an amino-functionalized polyhedral oligomeric silsesquioxane (POSS). Indeed, the developed approach is based on the potential reactivity of POSS amino group towards the polymer functionalities to produce an aminolysis reaction, which should promote the direct grafting of the silsesquioxane molecules on the polymer surface. Neat and treated films were studied by infrared spectroscopy and X-ray photoelectron spectroscopy, which proved the effectiveness of POSS grafting. Moreover, scanning electron microscopy measurements demonstrated the homogeneous distribution of Si on the film surface treated with the silsesquioxane. The influence of the film treatment on the surface wettability was evidenced by contact angle measurements. These findings demonstrated a relevant enhancement of the surface hydrophobicity, which increase turned out to depend on the conditions applied, as it increased by increasing the reaction temperature and the contact time. Finally, in order to evaluate the stability of neat and of the treated PLLA films the surface morphology of the samples treated with pH 7.4 buffer at 50 °C was studied.

## 1. Introduction

Polylactide (PLA), a biodegradable polyester, is one of the most interesting and sustainable substitutes of polymers from fossil resources [1]. Nevertheless, one of the major issues that reduces its exploitation in durable applications is its low hydrolytic stability compared with similar materials [2]. Indeed, PLA degrades by hydrolysis of the backbone ester groups, which reaction was demonstrated to be autocatalyzed by the polymer carboxylic acid end groups [3]. In particular, the degradation rate can be influenced by many features, such as the polymer chemical structure, molecular mass, molecular mass distribution, morphology, water diffusion rate into the matrix and water amount in the polymer [3]. The polymer decomposition is affected mainly by the polymer reactivity with water and availability of the ester groups to water and catalysts and it is accelerated by temperature [3,4,5]. In order to improve such features, several methods were developed based on the modification of the polymer structure [1], on the blending with other polymers [3], or with suitable fillers/nanofillers [6,7,8]. Although the above approaches were found to affect the polymer degradation, several drawbacks have to be taken into account for their use, including the change of the polymer features such as the transparency and the dispersibility of the additives. In this light, the development of easy approaches, using mild conditions, capable of enhancing the hydrolysis resistance of the polymer without affecting the bulk properties is a crucial issue for extending PLA exploitation.

On this basis, a valuable method should consider the change of the polymer surface without affecting its bulk. It is worth underling that the surface of PLA film was mainly modified by using plasma [9,10] and gas phase treatment [11]. In general, these methods were applied to enhance the PLA surface hydrophilicity, which modification turned out to increase the polymer hydrolytic degradation [8,9,10,11].

In our innovative approach, with the aim at limiting the hydrolytic degradation of PLA, an amino-functionalized polyhedral silsesquioxane (POSS) was used to graft to the surface of the polymer through an aminolysis reaction [12], thus modifying the surface properties. Indeed, POSS are soluble in common organic solvents and they are generally combined with polymers in order to obtain organic/inorganic systems with enhanced properties with respect to the matrix [13,14]. Generally, in order to produce polymer/POSS systems, melt or solvent blending were applied, by using a solvent capable of solubilizing both the silsesquioxane and the polymer in the case of the latter method [15]. In our approach, the polymer film is simply exposed to a solution of POSS to obtain a heterogenous reaction on the polymer surface. Both the neat and treated films were characterized by using Fourier Transform Infrared Spectroscopy (FTIR), X-ray Photoelectron Spectroscopy (XPS), Field Emission Scanning Electron Microscopy (FE-SEM), Differential Scanning Calorimetry (DSC), ThermoGravimetric Analysis (TGA) and contact angle measurements, while the hydrolytic degradation was followed by monitoring the film morphology over time.

## 2. Experimental

Poly(l-lactide) (PLLA) was purchased from Nature Works (BV, Naarden, The Netherlands), grade 2002D, Mn = 100,000 g/mol, with a residual monomer content less than 0.3% by mass, while aminopropyl heptaisobutyl-POSS (Figure S1, referred as POSS-NH_2_ from now on) was obtained from Hybrid Plastics (Hattiesburg, MS, USA). Hexane and dichloromethane (Sigma-Aldrich, Milano, Italy) were used without further purification. PLLA was dissolved in dichloromethane (concentration of 2 g/dL), cast on a glass Petri dish and it was allowed to air-dry. Then, with the aim at completely removing the solvent, the resulting films were dried in vacuum for 4 h at 40 °C and 4 h at 80 °C. Finally, the transparent films, which were formed on the dish with thickness of about 100 µm, were peeled off. The films were cut into squares of size 2 × 2 cm^2^ and were dipped in 20 mL of a solution of POSS-NH_2_ in hexane (2% *w*/*w*) by applying different times (4 and 8 h) and temperatures (40 °C and 60 °C). The above solvent was chosen on the basis of its capability of dissolving the silsesquioxane but not the polymer. The treated film was then washed with 20 mL of fresh hexane for one hour at the same temperature applied in the reaction and under magnetic stirring and with another 20 mL of fresh hexane overnight at room temperature. At the end, the film was allowed to dry in air and underwent the same thermal treatment as that used for the neat PLLA film, namely 4 h at 40 °C and 4 h at 80 °C. The samples were defined by indicating in the code the treatment time and temperature (as an example: PLLA_POSS_4_40 indicates a film treated with POSS-NH_2_ for 4 h at 40 °C).

A field emission scanning electron microscope (Supra 40 VP from Zeiss, Jena, Germany), holding a backscattered electron detector, was used to examine the developed material morphologies. The films were submerged in liquid nitrogen (30 min) and then they were fractured cryogenically. A sputter coater (Polaron E5100 by Quorum Technologies Ltd, Laughton, UK) was used to thinly sputter-coat the films with carbon. A Mettler-Toledo (Greifensee, Switzerland) TGA 1 thermogravimetric analyzer was applied, under a flow of nitrogen of 80 mL/min, between 25 and 800 °C, 20 °C/min heating rate, to study the thermal decomposition of the neat PLLA and of the treated films. Volatilization onset temperatures (T_onset_) were taken at 3% weight loss and temperatures for maximum volatilization rate (T_max_) were taken from at the maximum of derivative weight plot. Both T_onset_ and T_max_ are typically reproducible to ±3 °C. IR spectra were recorded by means of an IFS66 spectrometer by Bruker (Milano, Italy) considering a spectral range 400–4000 cm^−1^. Differential scanning calorimetric analysis was performed between 25 and 250 °C, at 10 °C/min heating and cooling rates, under a continuous nitrogen purge by using a DSC1 STARe calorimetric apparatus by Mettler (Greifensee, Switzerland). Glass transition temperatures (T_g_) were taken at midpoint of the transition on second heating plots, while cold crystallization temperatures (T_cc_) and melting temperatures were taken (T_m_) were taken as peak values on the second heating plot. T_g_, T_cc_ and T_m_ are typically reproducible to ±1°. Contact angle experiments were carried out at room temperature by means of an Attension contact angle meter and by exploiting pure water as probe liquid. In order to evaluate the film resistance to hydrolysis, small pieces of PLLA films (area of 1 × 1 cm^2^), which were previously dried overnight, were dipped into 10 mL of 0.1 M phosphate buffer solution (pH = 7.4) at 50 °C. The morphology of the degraded films was evaluated by FE-SEM analysis.

XPS measurements were accomplished by using a VersaProbe5000 by Physical Electronics (Chanhassen, MN, USA) equipped with a monochromatic Al source and a hemispherical analyzer. Both survey scans and high-resolution spectra were recorded by using a spot size of 100 µm. In order to eliminate the adsorbed molecules, the films were kept under vacuum for 15 h prior to the tests. A Shirley background function was exploited to adjust the spectra background. The curve fitting was accomplished by using a Gaussian (80%)–Lorentzian (20%) peak shape by minimizing the total square-error fit.

## 3. Results and Discussion

**Figure 1:**
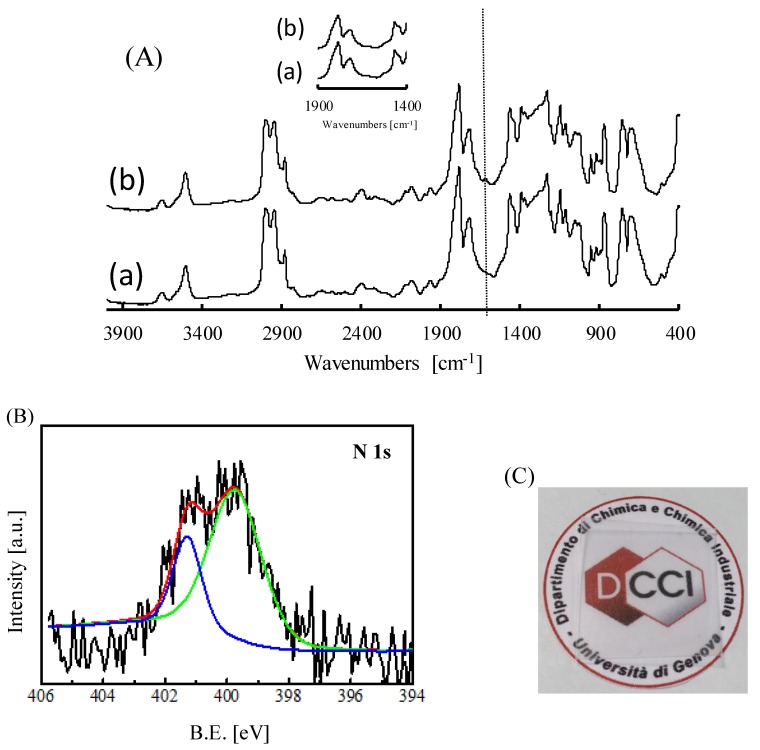
(**A**) FTIR spectra of (a) neat PLLA and (b) PLLA_8_60 film, (**B**) XPS spectrum of PLLA_8_60 film and (**C**) photo of PLLA_8_60 film.

The characteristics of the films treated with POSS-NH_2_ were compared with those of the neat PLLA films by investigating the influence of temperature (T_c_) and contact time (t_c_) on the material final features. At first, the occurrence of the reaction was studied by means of infrared spectroscopy. Figure 1A compares the FTIR spectrum of the neat PLLA film, with that of a film treated with POSS-NH_2_ at 60 °C for 8 h (PLLA_8_60). For the former sample, typical bands for PLA [2] were detected. In the treated film, together with the typical bands of PLLA spectrum, a new band at ca. 1600 cm^−1^ and a shoulder at ca. 1650 cm^−1^ (insert of Figure 1A and Figure S2A) appear, which can be ascribed to amide group formation [12]. Moreover, in the spectrum of the treated film, a slight deformation of the band at ca. 1080 cm^−1^ is visible—this change might be related with the presence of the silsesquioxane on the surface (Figure S2B). Indeed, a strong signal is present in this region in the spectrum of POSS [14], which belongs to the stretching of Si-O. Despite no strict correlation between the intensity of the above signals with contact time and the temperature was found, in the case of the samples exposed to the silesquioxane for a lower t_c_, namely PLLA_POSS_4_40 and PLLA_POSS_4_60, the band at 1600 cm^−1^ is barely visible. XPS measurements were performed to further corroborate these findings. The survey scans indicated the presence of Si in the treated samples, with a concentration around 5% in the films PLLA_POSS_8_40 and PLLA_POSS_8_60. Moreover, considering the chemical environment of N atoms, while POSS-NH_2_ (Figure S3), as previously reported [16], was found to hold a single N 1s peak centered at (399.7 ± 0.2) eV, which is ascribable to the presence of –NH_2_ functionalities, the XPS spectrum of PLLA_POSS_8_60 showed two N species: one, centered at (399.8 ± 0.2) eV, and the second, centered at (401.6 ± 0.2) eV (Figure 1B). All the other treated films showed a similar behaviour. This finding demonstrates the modification of N chemical environment, which can be associated to the reaction of POSS-NH_2_.

**Figure 2:**
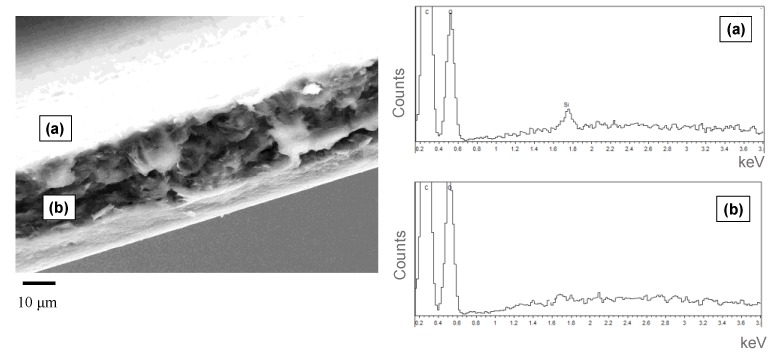
(left) FE-SEM of PLLA_POSS_8_60 film cross-section and (right) EDS analyses of the surface (point a) and cross-section (point b).

The surface and the cross-sections of the treated films were analysed by FE-SEM measurements coupled with Energy-Dispersive X-ray Spectroscopy (EDS) analysis, considering in particular Si dispersion, related to POSS distribution. Figure 2 shows a micrograph of the film cross-section, along with the elemental analysis. Indeed, while EDS measurements evidenced the presence of Si on the surface, whose concentration was found to be the same in the various analyzed points, in the cross section the above element was not detectable. This finding seems to evidence once again the deposition of POSS on the surface, which, as proved by FTIR and XPS measurements, was found to be covalently linked to the surface (reaction scheme reported in Figure S4).

It is worth underlining that despite the silsesquioxane deposition, the film appears to be transparent (Figure 1C), which property results to be essential for the real application of the material.

The thermal properties of the films were analyzed by DSC and TGA measurements. While DSC results evidenced a scarce influence of POSS deposition on PLLA crystallization (Table S1a), TGA measurements demonstrated a modification of the treated film thermal stability (Table S1b). As shown in Table S1b, both the onset degradation temperature (T_onset_) and the temperature corresponding maximum weight loss rate (T_max_) turned out to increase by increasing the contact time and temperature applied in the treatment of the films with POSS-NH_2_, the difference of T_max_ being around 10 °C for the sample PLLA_POSS_8_60. It is worth underlining that the influence of POSS on the degradation temperature of nanocomposites was widely studied and its specific effect was related to the formation of a silica layer on the surface of the polymer, behaving as a barrier and limiting the material decomposition [17].

**Figure 3:**
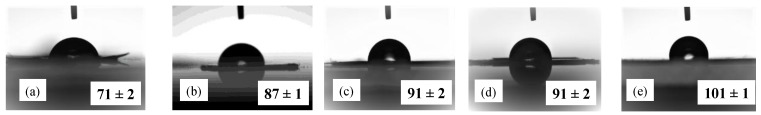
Water droplet placed on: (**a**) neat PLLA film, (**b**) PLLA_POSS_4_40 film, (**c**) PLLA_POSS_8_40 film, (**d**) PLLA_POSS_4_60 film and (**e**) PLLA_POSS_8_60 film.

In order to analyze the effect of the film treatment on the surface wettability, contact angle measurements were carried out. As previously mentioned, among the various factors affecting the decomposition of PLA (which occurs through hydrolysis of the backbone ester groups), the polymer reactivity with water and the accessibility of its ester groups to water were found to strongly determine the polymer degradation [4]. On this basis, considering that a modification of the surface wettability can lead to a change of the polymer hydrolytic decomposition, it is possible to infer that contact angle measurements can give significant information on the material behavior. In Figure 3 and Table S1b the contact angles measurements are given as a function of the reaction conditions, namely the tc and Tc. The untreated PLLA film was found to be characterized by a contact angle of 71° ± 2°, which is a value similar to that reported in the literature for neat PLA [18]. It is clear that the treatment with the silsesquioxane led to an increase of the contact angle, which was found to be incrementally affected by the applied conditions, as this value increased by increasing contact time and the temperature. In particular, the contact angles of the film treated for 8 h and 60 °C, PLLA_POSS_8_60, reached ca. 100°, which value proves the formation of a hydrophobic surface or might be related to an increase of the surface roughness. The influence of silsesquioxanes on surface properties was previously demonstrated for other POSS/polymer systems. Misra et al. [19] found for PP/octaisobutyl-POSS nanocomposites an increase of surface hydrophobicity with respect to the neat polymer matrix, while in a previous work of ours, the increment of contact angle was obtained for nanostructured films based on poly(styrene-co-maleic anhydride) and POSS [16]. These latter results were explained by considering the effect of the hydrophobic groups, linked to the silsesquioxane structure and the enhancement of surface roughness of the nanocomposite films. Also in our case, the increase of contact angle can be attributed to the hydrophobicity of POSS molecules, which, as previously demonstrated, turned out to be grafted to the polymer surface.

The decomposition behaviour of the PLA-based films was investigated by analyzing the macroscopic and microscopic morphology of films, both neat and treated with POSS-NH_2_, which were put in contact with water at 50 °C. The evaluation of the degradation process by measuring the weight of the samples turned out to be difficult, as mainly for the neat PLLA films, a relevant loss of integrity appears already after 20 days. The photos, reported in Figure S5, compared the former film with that treated with POSS-NH_2_, which were put in contact with the buffer for four weeks at 50 °C. It is clear that the neat PLLA film did not maintain its dimensional integrity, while the treated one showed a higher stability.

**Figure 4:**
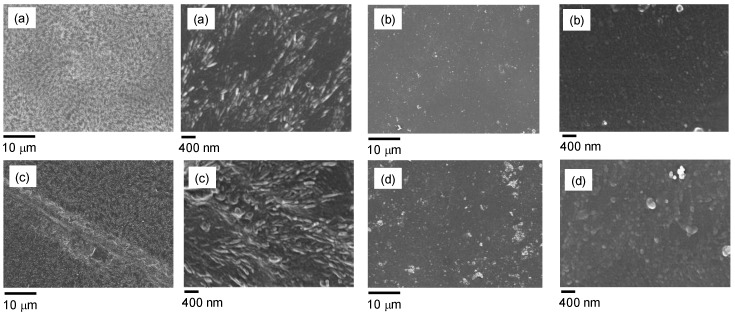
FE-SEM micrographs of: (**a**) neat PLLA film treated with the phosphate buffer solution at 50 °C for two weeks (left image at lower magnification, right image at higher magnification), (**b**) PLLA_POSS_8_60 film treated with the phosphate buffer solution at 50 °C for two weeks (left image at lower magnification, right image at higher magnification), (**c**) neat PLLA film treated with the phosphate buffer solution at 50 °C for four weeks (left image at lower magnification, right image at higher magnification), (**d**) PLLA_POSS_8_60 film treated with the phosphate buffer solution at 50 °C for four weeks (left image at lower magnification, right image at higher magnification).

The morphology of the degraded films was analyzed by means of FE-SEM measurements. Figure 4 shows the micrographs of the surfaces of the samples PLLA and PLLA_POSS_8_60, which were put in contact with the buffer at 50 °C for two or four weeks. While the surface of the neat films appeared to be homogeneous and uniform, significant changes were visible in the films which underwent a degradation process. The neat PLLA films showed the morphology typical of banded spherulites, which gave evidence of the presence of PLA crystallites [20], while increasing the contact time the surface roughness and cracks seemed to increase (Figure 4c). This phenomenon is explained by considering that the hydrolysis of the film, which involves the amorphous fraction of the polymer and produces short chains, easily solubilized in water, makes the crystalline structure become visible. In the case of the POSS-treated samples, although the degradation led to an increase of the surface roughness, the spherulite morphology was not visible. This finding demonstrates that the degradation mechanism of the films is significantly affected by the presence of the silsesquioxane. It is possible to infer that the POSS surface grafting limits the degradation of the polymer amorphous fraction, thus leading to an enhancement of the material resistance.

## 4. Conclusions

This work demonstrated the effectiveness of the surface grafting of an amino-functionalized polyhedral oligomeric silsesquioxanes on improving the resistance to the hydrolytic degradation of poly(l-lactide) films. The developed method, which is simple and easily scalable, is based the aminolysis reaction between the amino group of the silsesquioxane and the polymer functionalities. The characterization measurements gave evidence of the POSS grafting occurrence as well as the increment of the surface hydrophilicity, which limited the film’s hydrolytic degradation.

## Supplementary Materials

The following are available online at https://www.mdpi.com/2079-4991/9/8/1144/s1, Figure S1: Structure of amino-functionalized POSS, Figure S2: additional FTIR spectra, Figure S3: XPS for POSS-NH_2_, Figure S4: Reaction mechanism for POSS-NH_2_ on PLLA, Figure S5: photographs for PLLA and PLLA treated with POSS-NH_2_ after 4 weeks hydrolysis, Table S1a: crystallization results, Table S1b: Thermal stability and contact angle results.
