# Supplementary material for: Polyhedral Oligomeric Silsesquioxane (POSS) Surface Grafting: A Novel Method to Enhance Polylactide Hydrolysis Resistance
Source: Nanomaterials (Basel). 2019 Aug 9;9(8):1144. doi: 10.3390/nano9081144 (PMC6723249; doi:10.3390/nano9081144)
Supplement: Supplementary file 1 [file nanomaterials-09-01144-s001.pdf]

Communication

Polyhedral Oligomeric Silsesquioxane (POSS) Surface Grafting: A Novel Method to Enhance Polylactide Hydrolysis Resistance

Kun Li [1], Samuele Colonna [2], Alberto Fina [2] and Orietta Monticelli [1,*]

[1] Dipartimento di Chimica e Chimica Industriale, Università di Genova, Via Dodecaneso, 31, 16146 Genova, Italy

[2] Dipartimento di Scienza Applicata e Tecnologia, Politecnico di Torino- Alessandria campus, viale Teresa Michel, 5, 15121 Alessandria, Italy

* Correspondence: orietta.monticelli@unige.it

Received: 2 July 2019; Accepted: 6 August 2019; Published: date

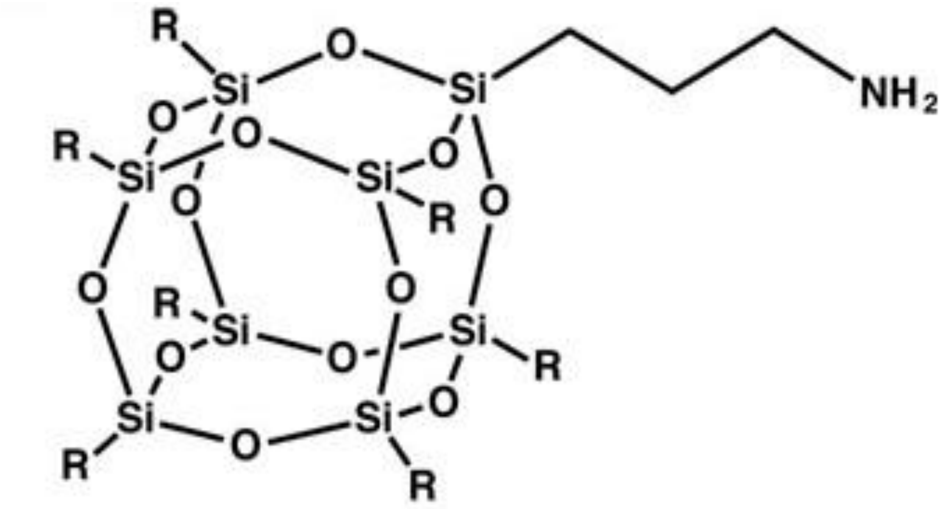

Figure S1. Aminopropyl heptaisobutyl POSS (POSS-NH_2).

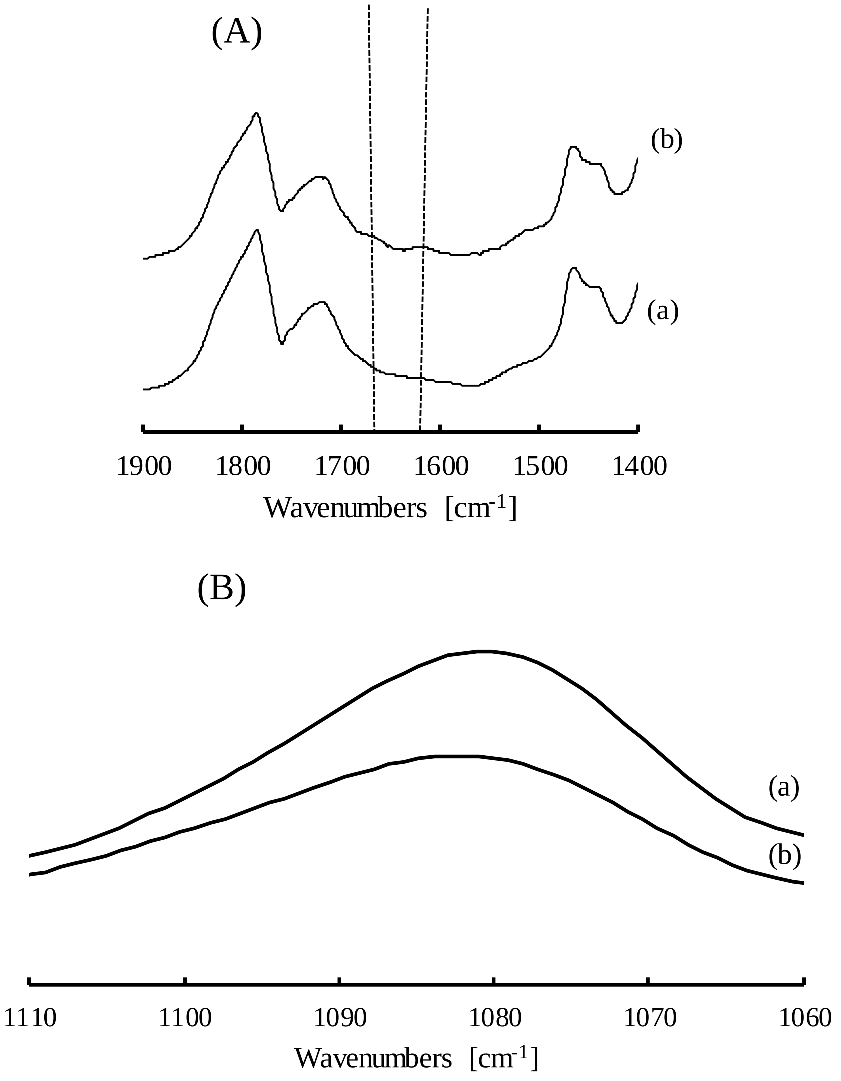

Figure S2. (**A**) FTIR spectra of: (a) PLLA neat film and (b) PLLA_POSS_8_60 film in the range 1900–1400 cm^{-1}, (**B**) FTIR spectra of: (a) PLLA neat film and (b) PLLA_POSS_8_60 film in the range 1110–1060 cm^{-1}.

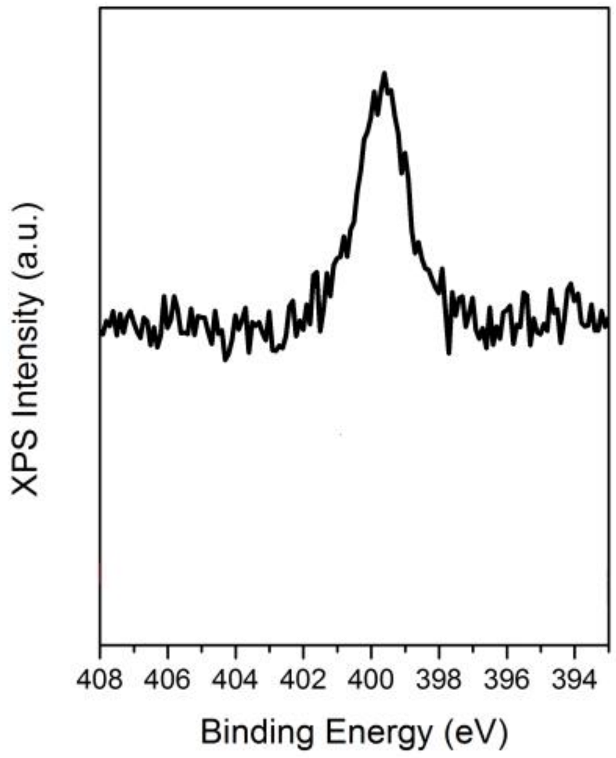

Figure S3. XPS spectrum collected on POSS-NH_2 powders. The data are shown in the energy region typical for N 1s photoelectrons after subtraction of Shirley-type background. Data are shown after normalization.

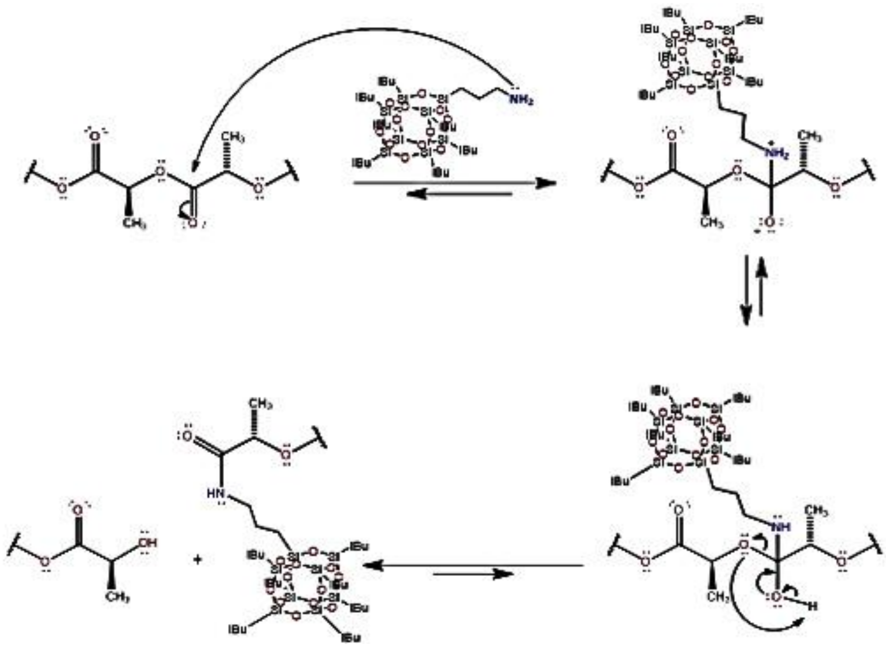

Figure S4. Reaction mechanism of the reaction between POSS-NH_2 and PLLA.

Table S1a. DSC results of neat PLLA and of treated films (second heating).

Sample code	T_g (°C)	T_{cc} (°C)	T_m (°C)	ΔH_{cc} (J/g)	ΔH_m (J/g)
PLLA	60	130	153	5	6
PLLA_POSS_4_40	61	133	154	5	6
PLA/POSS_8_40	60	131	154	4	6
PLLA_POSS_4_60	60	130	153	5	6
PLLA_POSS_8_60	348	130	153	5	6

T_g: glass transition temperature, T_{cc}: cold crystallization temperature, T_m: melting temperature, ΔH_{cc}: enthalpy of the cold crystallization, ΔH_m: melting enthalpy.

Table S1b Characterization results of neat PLLA and of treated films.

Sample code	T_{onset} (°C)	T_{max} (°C)	Contact angles (°)
PLLA	329	367	71 ±2
PLLA_POSS_4_40	331	369	87 ±1
PLA/POSS_8_40	334	371	91 ±2
PLLA_POSS_4_60	341	374	91 ±2
PLLA_POSS_8_60	348	378	101 ±1

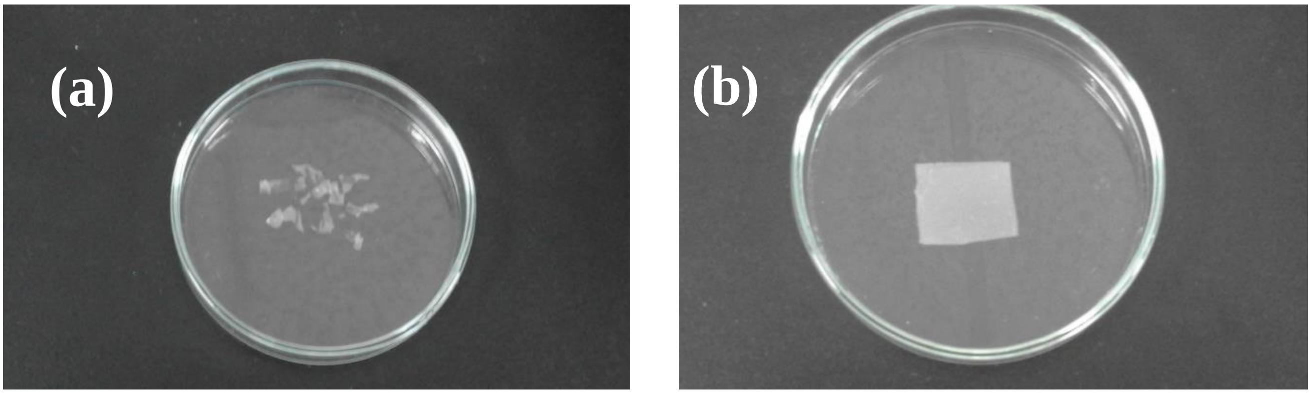

Figure S5. Photos of (**a**) PLLA film and (**b**) PLLA_POSS_8_60 film after being in contact with the buffer for 4 weeks at 50 °C.
